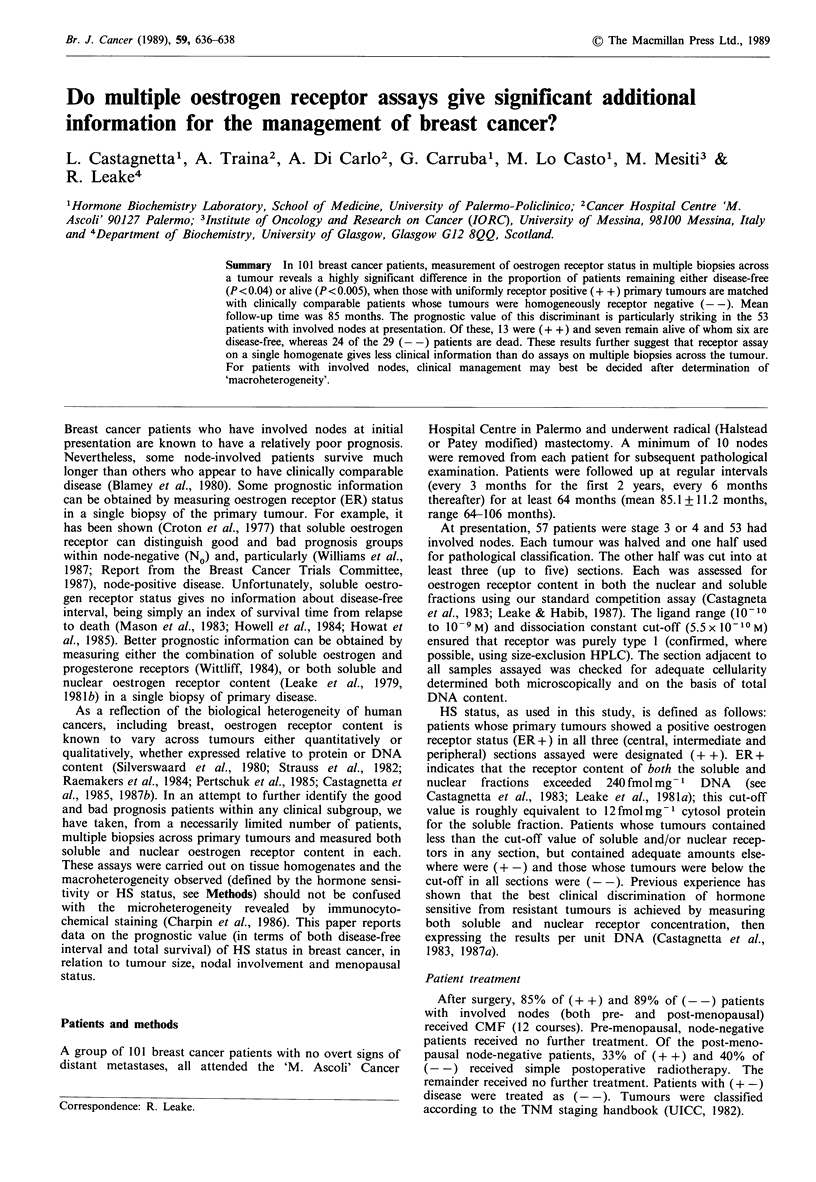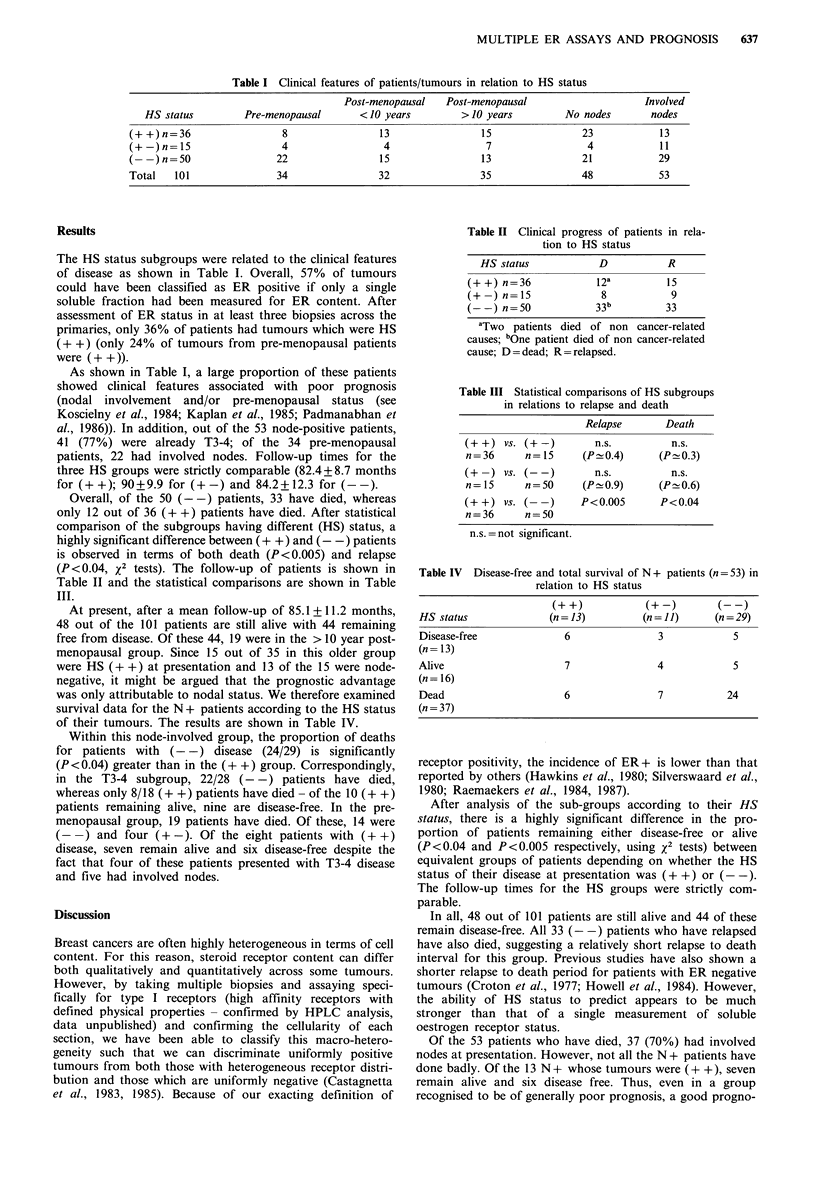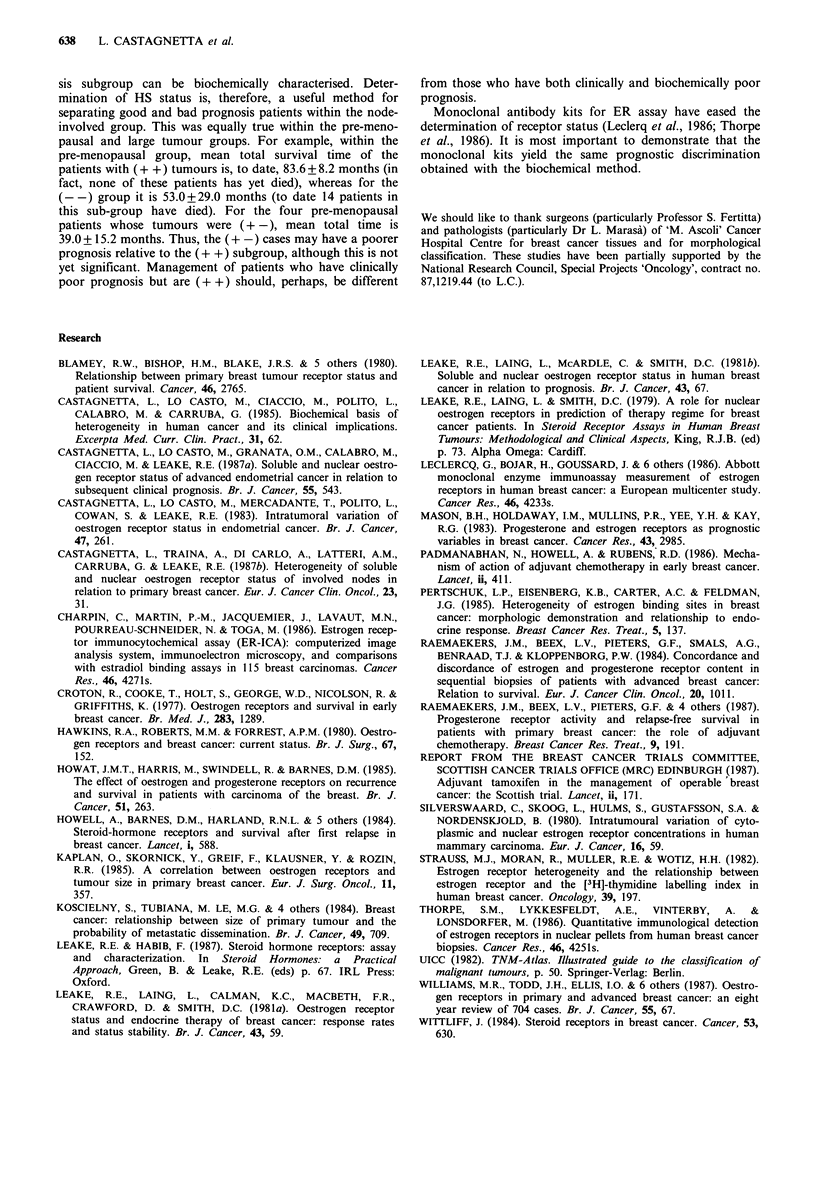# Do multiple oestrogen receptor assays give significant additional information for the management of breast cancer?

**DOI:** 10.1038/bjc.1989.129

**Published:** 1989-04

**Authors:** L. Castagnetta, A. Traina, A. Di Carlo, G. Carruba, M. Lo Casto, M. Mesiti, R. Leake

**Affiliations:** Hormone Biochemistry Laboratory, School of Medicine, University of Palermo-Policlinico, Italy.

## Abstract

In 101 breast cancer patients, measurement of oestrogen receptor status in multiple biopsies across a tumour reveals a highly significant difference in the proportion of patients remaining either disease-free (P less than 0.04) or alive (P less than 0.005), when those with uniformly receptor positive (++) primary tumours are matched with clinically comparable patients whose tumours were homogeneously receptor negative (--). Mean follow-up time was 85 months. The prognostic value of this discriminant is particularly striking in the 53 patients with involved nodes at presentation. Of these, 13 were (++) and seven remain alive of whom six are disease-free, whereas 24 of the 29 (--) patients are dead. These results further suggest that receptor assay on a single homogenate gives less clinical information than do assays on multiple biopsies across the tumour. For patients with involved nodes, clinical management may best be decided after determination of 'macroheterogeneity'.


Br. J. Cancer (1989), 59, 636–638 © The Macmillan Press Ltd., 1989

### Do multiple oestrogen receptor assays give significant additional information for the management of breast cancer?

L. Castagnetta[1], A. Traina[2], A. Di Carlo[2], G. Carruba[1], M. Lo Casto[1], M. Mesiti[3] & R. Leake[4]

[1]*Hormone Biochemistry Laboratory, School of Medicine, University of Palermo-Policlinico;* [2]*Cancer Hospital Centre 'M. Ascoli' 90127 Palermo;* [3]*Institute of Oncology and Research on Cancer (IORC), University of Messina, 98100 Messina, Italy and* [4]*Department of Biochemistry, University of Glasgow, Glasgow G12 8QQ, Scotland.*

**Summary** In 101 breast cancer patients, measurement of oestrogen receptor status in multiple biopsies across a tumour reveals a highly significant difference in the proportion of patients remaining either disease-free ($P<0.04$) or alive ($P<0.005$), when those with uniformly receptor positive (+ +) primary tumours are matched with clinically comparable patients whose tumours were homogeneously receptor negative (− −). Mean follow-up time was 85 months. The prognostic value of this discriminant is particularly striking in the 53 patients with involved nodes at presentation. Of these, 13 were (+ +) and seven remain alive of whom six are disease-free, whereas 24 of the 29 (− −) patients are dead. These results further suggest that receptor assay on a single homogenate gives less clinical information than do assays on multiple biopsies across the tumour. For patients with involved nodes, clinical management may best be decided after determination of 'macroheterogeneity'.

Breast cancer patients who have involved nodes at initial presentation are known to have a relatively poor prognosis. Nevertheless, some node-involved patients survive much longer than others who appear to have clinically comparable disease (Blamey *et al.*, 1980). Some prognostic information can be obtained by measuring oestrogen receptor (ER) status in a single biopsy of the primary tumour. For example, it has been shown (Croton *et al.*, 1977) that soluble oestrogen receptor can distinguish good and bad prognosis groups within node-negative ($N_0$) and, particularly (Williams *et al.*, 1987; Report from the Breast Cancer Trials Committee, 1987), node-positive disease. Unfortunately, soluble oestrogen receptor status gives no information about disease-free interval, being simply an index of survival time from relapse to death (Mason *et al.*, 1983; Howell *et al.*, 1984; Howat *et al.*, 1985). Better prognostic information can be obtained by measuring either the combination of soluble oestrogen and progesterone receptors (Wittliff, 1984), or both soluble and nuclear oestrogen receptor content (Leake *et al.*, 1979, 1981*b*) in a single biopsy of primary disease.

As a reflection of the biological heterogeneity of human cancers, including breast, oestrogen receptor content is known to vary across tumours either quantitatively or qualitatively, whether expressed relative to protein or DNA content (Silverswaard *et al.*, 1980; Strauss *et al.*, 1982; Raemakers *et al.*, 1984; Pertschuk *et al.*, 1985; Castagnetta *et al.*, 1985, 1987*b*). In an attempt to further identify the good and bad prognosis patients within any clinical subgroup, we have taken, from a necessarily limited number of patients, multiple biopsies across primary tumours and measured both soluble and nuclear oestrogen receptor content in each. These assays were carried out on tissue homogenates and the macroheterogeneity observed (defined by the hormone sensitivity or HS status, see **Methods**) should not be confused with the microheterogeneity revealed by immunocytochemical staining (Charpin *et al.*, 1986). This paper reports data on the prognostic value (in terms of both disease-free interval and total survival) of HS status in breast cancer, in relation to tumour size, nodal involvement and menopausal status.

### Patients and methods

A group of 101 breast cancer patients with no overt signs of distant metastases, all attended the 'M. Ascoli' Cancer Hospital Centre in Palermo and underwent radical (Halstead or Patey modified) mastectomy. A minimum of 10 nodes were removed from each patient for subsequent pathological examination. Patients were followed up at regular intervals (every 3 months for the first 2 years, every 6 months thereafter) for at least 64 months (mean $85.1 \pm 11.2$ months, range 64–106 months).

At presentation, 57 patients were stage 3 or 4 and 53 had involved nodes. Each tumour was halved and one half used for pathological classification. The other half was cut into at least three (up to five) sections. Each was assessed for oestrogen receptor content in both the nuclear and soluble fractions using our standard competition assay (Castagneta *et al.*, 1983; Leake & Habib, 1987). The ligand range ($10^{-10}$ to $10^{-9}$ M) and dissociation constant cut-off ($5.5 \times 10^{-10}$ M) ensured that receptor was purely type 1 (confirmed, where possible, using size-exclusion HPLC). The section adjacent to all samples assayed was checked for adequate cellularity determined both microscopically and on the basis of total DNA content.

HS status, as used in this study, is defined as follows: patients whose primary tumours showed a positive oestrogen receptor status (ER+) in all three (central, intermediate and peripheral) sections assayed were designated (+ +). ER+ indicates that the receptor content of *both* the soluble and nuclear fractions exceeded 240 $\text{fmol mg}^{-1}$ DNA (see Castagnetta *et al.*, 1983; Leake *et al.*, 1981*a*); this cut-off value is roughly equivalent to 12 $\text{fmol mg}^{-1}$ cytosol protein for the soluble fraction. Patients whose tumours contained less than the cut-off value of soluble and/or nuclear receptors in any section, but contained adequate amounts elsewhere were (+ −) and those whose tumours were below the cut-off in all sections were (− −). Previous experience has shown that the best clinical discrimination of hormone sensitive from resistant tumours is achieved by measuring both soluble and nuclear receptor concentration, then expressing the results per unit DNA (Castagnetta *et al.*, 1983, 1987*a*).

### *Patient treatment*

After surgery, 85% of (+ +) and 89% of (− −) patients with involved nodes (both pre- and post-menopausal) received CMF (12 courses). Pre-menopausal, node-negative patients received no further treatment. Of the post-menopausal node-negative patients, 33% of (+ +) and 40% of (− −) received simple postoperative radiotherapy. The remainder received no further treatment. Patients with (+ −) disease were treated as (− −). Tumours were classified according to the TNM staging handbook (UICC, 1982).

Correspondence: R. Leake.

**Table I** Clinical features of patients/tumours in relation to HS status

| *HS status* | *Pre-menopausal* | *Post-menopausal <10 years* | *Post-menopausal >10 years* | *No nodes* | *Involved nodes* |
|---|---|---|---|---|---|
| (+ +) $n=36$ | 8 | 13 | 15 | 23 | 13 |
| (+ −) $n=15$ | 4 | 4 | 7 | 4 | 11 |
| (− −) $n=50$ | 22 | 15 | 13 | 21 | 29 |
| Total 101 | 34 | 32 | 35 | 48 | 53 |

### Results

The HS status subgroups were related to the clinical features of disease as shown in Table I. Overall, 57% of tumours could have been classified as ER positive if only a single soluble fraction had been measured for ER content. After assessment of ER status in at least three biopsies across the primaries, only 36% of patients had tumours which were HS (+ +) (only 24% of tumours from pre-menopausal patients were (+ +)).

As shown in Table I, a large proportion of these patients showed clinical features associated with poor prognosis (nodal involvement and/or pre-menopausal status (see Koscielny *et al.*, 1984; Kaplan *et al.*, 1985; Padmanabhan *et al.*, 1986)). In addition, out of the 53 node-positive patients, 41 (77%) were already T3-4; of the 34 pre-menopausal patients, 22 had involved nodes. Follow-up times for the three HS groups were strictly comparable (82.4±8.7 months for (+ +); 90±9.9 for (+ −) and 84.2±12.3 for (− −).

Overall, of the 50 (− −) patients, 33 have died, whereas only 12 out of 36 (+ +) patients have died. After statistical comparison of the subgroups having different (HS) status, a highly significant difference between (+ +) and (− −) patients is observed in terms of both death ($P<0.005$) and relapse ($P<0.04$, $\chi^2$ tests). The follow-up of patients is shown in Table II and the statistical comparisons are shown in Table III.

At present, after a mean follow-up of 85.1±11.2 months, 48 out of the 101 patients are still alive with 44 remaining free from disease. Of these 44, 19 were in the >10 year post-menopausal group. Since 15 out of 35 in this older group were HS (+ +) at presentation and 13 of the 15 were node-negative, it might be argued that the prognostic advantage was only attributable to nodal status. We therefore examined survival data for the N+ patients according to the HS status of their tumours. The results are shown in Table IV.

Within this node-involved group, the proportion of deaths for patients with (− −) disease (24/29) is significantly ($P<0.04$) greater than in the (+ +) group. Correspondingly, in the T3-4 subgroup, 22/28 (− −) patients have died, whereas only 8/18 (+ +) patients have died – of the 10 (+ +) patients remaining alive, nine are disease-free. In the pre-menopausal group, 19 patients have died. Of these, 14 were (− −) and four (+ −). Of the eight patients with (+ +) disease, seven remain alive and six disease-free despite the fact that four of these patients presented with T3-4 disease and five had involved nodes.

### Discussion

Breast cancers are often highly heterogeneous in terms of cell content. For this reason, steroid receptor content can differ both qualitatively and quantitatively across some tumours. However, by taking multiple biopsies and assaying specifically for type I receptors (high affinity receptors with defined physical properties – confirmed by HPLC analysis, data unpublished) and confirming the cellularity of each section, we have been able to classify this macro-heterogeneity such that we can discriminate uniformly positive tumours from both those with heterogeneous receptor distribution and those which are uniformly negative (Castagnetta *et al.*, 1983, 1985). Because of our exacting definition of receptor positivity, the incidence of ER+ is lower than that reported by others (Hawkins *et al.*, 1980; Silverswaard *et al.*, 1980; Raemaekers *et al.*, 1984, 1987).

**Table II** Clinical progress of patients in relation to HS status

| *HS status* | *D* | *R* |
|---|---|---|
| (+ +) $n=36$ | 12[a] | 15 |
| (+ −) $n=15$ | 8 | 9 |
| (− −) $n=50$ | 33[b] | 33 |

[a]Two patients died of non cancer-related causes; [b]One patient died of non cancer-related cause; D = dead; R = relapsed.

**Table III** Statistical comparisons of HS subgroups in relations to relapse and death

| | *Relapse* | *Death* |
|---|---|---|
| (+ +) *vs.* (+ −) $n=36$ $n=15$ | n.s. ($P\simeq0.4$) | n.s. ($P\simeq0.3$) |
| (+ −) *vs.* (− −) $n=15$ $n=50$ | n.s. ($P\simeq0.9$) | n.s. ($P\simeq0.6$) |
| (+ +) *vs.* (− −) $n=36$ $n=50$ | $P<0.005$ | $P<0.04$ |

n.s. = not significant.

**Table IV** Disease-free and total survival of N+ patients ($n=53$) in relation to HS status

| *HS status* | (+ +) ($n=13$) | (+ −) ($n=11$) | (− −) ($n=29$) |
|---|---|---|---|
| Disease-free ($n=13$) | 6 | 3 | 5 |
| Alive ($n=16$) | 7 | 4 | 5 |
| Dead ($n=37$) | 6 | 7 | 24 |

After analysis of the sub-groups according to their *HS status*, there is a highly significant difference in the proportion of patients remaining either disease-free or alive ($P<0.04$ and $P<0.005$ respectively, using $\chi^2$ tests) between equivalent groups of patients depending on whether the HS status of their disease at presentation was (+ +) or (− −). The follow-up times for the HS groups were strictly comparable.

In all, 48 out of 101 patients are still alive and 44 of these remain disease-free. All 33 (− −) patients who have relapsed have also died, suggesting a relatively short relapse to death interval for this group. Previous studies have also shown a shorter relapse to death period for patients with ER negative tumours (Croton *et al.*, 1977; Howell *et al.*, 1984). However, the ability of HS status to predict appears to be much stronger than that of a single measurement of soluble oestrogen receptor status.

Of the 53 patients who have died, 37 (70%) had involved nodes at presentation. However, not all the N+ patients have done badly. Of the 13 N+ whose tumours were (+ +), seven remain alive and six disease free. Thus, even in a group recognised to be of generally poor prognosis, a good prognosis subgroup can be biochemically characterised. Determination of HS status is, therefore, a useful method for separating good and bad prognosis patients within the node-involved group. This was equally true within the pre-menopausal and large tumour groups. For example, within the pre-menopausal group, mean total survival time of the patients with (+ +) tumours is, to date, $83.6 \pm 8.2$ months (in fact, none of these patients has yet died), whereas for the (− −) group it is $53.0 \pm 29.0$ months (to date 14 patients in this sub-group have died). For the four pre-menopausal patients whose tumours were (+ −), mean total time is $39.0 \pm 15.2$ months. Thus, the (+ −) cases may have a poorer prognosis relative to the (+ +) subgroup, although this is not yet significant. Management of patients who have clinically poor prognosis but are (+ +) should, perhaps, be different from those who have both clinically and biochemically poor prognosis.

Monoclonal antibody kits for ER assay have eased the determination of receptor status (Leclerq *et al.*, 1986; Thorpe *et al.*, 1986). It is most important to demonstrate that the monoclonal kits yield the same prognostic discrimination obtained with the biochemical method.

We should like to thank surgeons (particularly Professor S. Fertitta) and pathologists (particularly Dr L. Marasà) of 'M. Ascoli' Cancer Hospital Centre for breast cancer tissues and for morphological classification. These studies have been partially supported by the National Research Council, Special Projects 'Oncology', contract no. 87,1219.44 (to L.C.).

### Research

BLAMEY, R.W., BISHOP, H.M., BLAKE, J.R.S. & 5 others (1980). Relationship between primary breast tumour receptor status and patient survival. *Cancer*, **46**, 2765.

CASTAGNETTA, L., LO CASTO, M., CIACCIO, M., POLITO, L., CALABRO, M. & CARRUBA, G. (1985). Biochemical basis of heterogeneity in human cancer and its clinical implications. *Excerpta Med. Curr. Clin. Pract.*, **31**, 62.

CASTAGNETTA, L., LO CASTO, M., GRANATA, O.M., CALABRO, M., CIACCIO, M. & LEAKE, R.E. (1987*a*). Soluble and nuclear oestrogen receptor status of advanced endometrial cancer in relation to subsequent clinical prognosis. *Br. J. Cancer*, **55**, 543.

CASTAGNETTA, L., LO CASTO, M., MERCADANTE, T., POLITO, L., COWAN, S. & LEAKE, R.E. (1983). Intratumoral variation of oestrogen receptor status in endometrial cancer. *Br. J. Cancer*, **47**, 261.

CASTAGNETTA, L., TRAINA, A., DI CARLO, A., LATTERI, A.M., CARRUBA, G. & LEAKE, R.E. (1987*b*). Heterogeneity of soluble and nuclear oestrogen receptor status of involved nodes in relation to primary breast cancer. *Eur. J. Cancer Clin. Oncol.*, **23**, 31.

CHARPIN, C., MARTIN, P.-M., JACQUEMIER, J., LAVAUT, M.N., POURREAU-SCHNEIDER, N. & TOGA, M. (1986). Estrogen receptor immunocytochemical assay (ER-ICA): computerized image analysis system, immunoelectron microscopy, and comparisons with estradiol binding assays in 115 breast carcinomas. *Cancer Res.*, **46**, 4271s.

CROTON, R., COOKE, T., HOLT, S., GEORGE, W.D., NICOLSON, R. & GRIFFITHS, K. (1977). Oestrogen receptors and survival in early breast cancer. *Br. Med. J.*, **283**, 1289.

HAWKINS, R.A., ROBERTS, M.M. & FORREST, A.P.M. (1980). Oestrogen receptors and breast cancer: current status. *Br. J. Surg.*, **67**, 152.

HOWAT, J.M.T., HARRIS, M., SWINDELL, R. & BARNES, D.M. (1985). The effect of oestrogen and progesterone receptors on recurrence and survival in patients with carcinoma of the breast. *Br. J. Cancer*, **51**, 263.

HOWELL, A., BARNES, D.M., HARLAND, R.N.L. & 5 others (1984). Steroid-hormone receptors and survival after first relapse in breast cancer. *Lancet*, **i**, 588.

KAPLAN, O., SKORNICK, Y., GREIF, F., KLAUSNER, Y. & ROZIN, R.R. (1985). A correlation between oestrogen receptors and tumour size in primary breast cancer. *Eur. J. Surg. Oncol.*, **11**, 357.

KOSCIELNY, S., TUBIANA, M. LE, M.G. & 4 others (1984). Breast cancer: relationship between size of primary tumour and the probability of metastatic dissemination. *Br. J. Cancer*, **49**, 709.

LEAKE, R.E. & HABIB, F. (1987). Steroid hormone receptors: assay and characterization. In *Steroid Hormones: a Practical Approach*, Green, B. & Leake, R.E. (eds) p. 67. IRL Press: Oxford.

LEAKE, R.E., LAING, L., CALMAN, K.C., MACBETH, F.R., CRAWFORD, D. & SMITH, D.C. (1981*a*). Oestrogen receptor status and endocrine therapy of breast cancer: response rates and status stability. *Br. J. Cancer*, **43**, 59.

LEAKE, R.E., LAING, L., McARDLE, C. & SMITH, D.C. (1981*b*). Soluble and nuclear oestrogen receptor status in human breast cancer in relation to prognosis. *Br. J. Cancer*, **43**, 67.

LEAKE, R.E., LAING, L. & SMITH, D.C. (1979). A role for nuclear oestrogen receptors in prediction of therapy regime for breast cancer patients. In *Steroid Receptor Assays in Human Breast Tumours: Methodological and Clinical Aspects*, King, R.J.B. (ed) p. 73. Alpha Omega: Cardiff.

LECLERCQ, G., BOJAR, H., GOUSSARD, J. & 6 others (1986). Abbott monoclonal enzyme immunoassay measurement of estrogen receptors in human breast cancer: a European multicenter study. *Cancer Res.*, **46**, 4233s.

MASON, B.H., HOLDAWAY, I.M., MULLINS, P.R., YEE, Y.H. & KAY, R.G. (1983). Progesterone and estrogen receptors as prognostic variables in breast cancer. *Cancer Res.*, **43**, 2985.

PADMANABHAN, N., HOWELL, A. & RUBENS, R.D. (1986). Mechanism of action of adjuvant chemotherapy in early breast cancer. *Lancet*, **ii**, 411.

PERTSCHUK, L.P., EISENBERG, K.B., CARTER, A.C. & FELDMAN, J.G. (1985). Heterogeneity of estrogen binding sites in breast cancer: morphologic demonstration and relationship to endocrine response. *Breast Cancer Res. Treat.*, **5**, 137.

RAEMAEKERS, J.M., BEEX, L.V., PIETERS, G.F., SMALS, A.G., BENRAAD, T.J. & KLOPPENBORG, P.W. (1984). Concordance and discordance of estrogen and progesterone receptor content in sequential biopsies of patients with advanced breast cancer: Relation to survival. *Eur. J. Cancer Clin. Oncol.*, **20**, 1011.

RAEMAEKERS, J.M., BEEX, L.V., PIETERS, G.F. & 4 others (1987). Progesterone receptor activity and relapse-free survival in patients with primary breast cancer: the role of adjuvant chemotherapy. *Breast Cancer Res. Treat.*, **9**, 191.

REPORT FROM THE BREAST CANCER TRIALS COMMITTEE, SCOTTISH CANCER TRIALS OFFICE (MRC) EDINBURGH (1987). Adjuvant tamoxifen in the management of operable breast cancer: the Scottish trial. *Lancet*, **ii**, 171.

SILVERSWAARD, C., SKOOG, L., HULMS, S., GUSTAFSSON, S.A. & NORDENSKJOLD, B. (1980). Intratumoural variation of cytoplasmic and nuclear estrogen receptor concentrations in human mammary carcinoma. *Eur. J. Cancer*, **16**, 59.

STRAUSS, M.J., MORAN, R., MULLER, R.E. & WOTIZ, H.H. (1982). Estrogen receptor heterogeneity and the relationship between estrogen receptor and the [$^3$H]-thymidine labelling index in human breast cancer. *Oncology*, **39**, 197.

THORPE, S.M., LYKKESFELDT, A.E., VINTERBY, A. & LONSDORFER, M. (1986). Quantitative immunological detection of estrogen receptors in nuclear pellets from human breast cancer biopsies. *Cancer Res.*, **46**, 4251s.

UICC (1982). *TNM-Atlas. Illustrated guide to the classification of malignant tumours*, p. 50. Springer-Verlag: Berlin.

WILLIAMS, M.R., TODD, J.H., ELLIS, I.O. & 6 others (1987). Oestrogen receptors in primary and advanced breast cancer: an eight year review of 704 cases. *Br. J. Cancer*, **55**, 67.

WITTLIFF, J. (1984). Steroid receptors in breast cancer. *Cancer*, **53**, 630.